# Spanish version of the Pediatric Anesthesia Emergence Delirium scale: translation and cross-cultural adaptation

**DOI:** 10.1186/s12871-022-01893-1

**Published:** 2022-11-14

**Authors:** Valeria Epulef, Sergio Muñoz, Ana María Alarcón, Manuel Vial

**Affiliations:** 1 Department of Surgery, Traumatology and Anesthesiology, Medicine Faculty, Universidad de La Frontera, Temuco, Chile; 2 CIGES, Medicine Faculty, Universidad de La Frontera, Temuco, Chile; 3 Department of Public Health, Medicine Faculty, Universidad de La Frontera, Temuco, Chile

**Keywords:** Emergence delirium, Anesthesia recovery period, Pediatrics, Ambulatory surgical procedures, Postanesthesia nursing

## Abstract

**Background:**

Emergence delirium (ED) is a mental disturbance in children during recovery from general anaesthesia. The Pediatric Anesthesia Emergence Delirium (PAED) scale is the only validated scale that assesses ED in paediatric patients undergoing general anaesthesia. The aim of this study was the translation and cross-cultural adaptation of the PAED scale into Spanish (Chile).

**Methods:**

A five-stage translation and cross-cultural adaptation process was carried out. The reliability of the Spanish version of the PAED scale was evaluated in paediatric patients independently by a set of two raters (anaesthesiologists or postanaesthesia care unit nurses) in the postanaesthetic period after major outpatient surgery. ED was defined by a cut-off level of ≥ 10 points on the PAED scale.

**Results:**

The PAED scale was evaluated in 353 consecutive children. Patients had a mean age of 7.4 ± 3.22 years. The preoperative ASA Physical Status class was 62%, 37%, and 1% (ASA class I, II and III, respectively). The distribution of patients by service was as follows: 45% of patients underwent paediatric surgery; 33% underwent otorhinolaryngological surgery; 11% underwent orthopaedic surgery; 10% underwent ophthalmological surgery; and 1% underwent other types of surgery. The interrater agreement ranged from 96.9% to 97.9%, with Kappa values ranging from 0.59 to 0.79. The Cronbach’s alpha value was 0.91. The ED global incidence was 9.1% and was higher in the younger age groups (3–10 years).

**Conclusions:**

The translated and cross-culturally adapted Spanish version of the PAED scale is a reliable instrument to measure ED in the postanaesthetic period in Chilean children.

**Supplementary Information:**

The online version contains supplementary material available at 10.1186/s12871-022-01893-1.

## Background

Paediatric emergence delirium (ED) is a cluster of behavioural disturbances, including restlessness, excitation, inconsolable crying, and delusions, that can occur in the early postanaesthetic period [1]. The incidence of ED in paediatric patients ranges from 5 to 50% [2–4], and ED occurs more frequently in preschool children [5]. ED involves short-lived episodes that may lead to self-injury, delayed discharge, emotional distress for family members and an increased workload for postanaesthesia care unit (PACU) nurses [6]. ED must be measured to assess, treat and understand it and to develop prevention strategies [7]. 

Many scales have been used to measure ED in paediatric patients, but only one scale was developed to specifically assess ED in young children through a proper validation process: the PAED scale [8]. The PAED scale has five items scored from 0 to 4 (Additional file 1) and has been widely used and adapted to several scenarios [1, 7, 9–11], but it has not been validated for use in Spanish. The aim of this study was the translation and cross-cultural adaptation of the PAED scale into Spanish (Chile).

## Methods

This observational study was conducted in the Hospital Dr. Hernán Henríquez Aravena, Temuco, Chile, between July 2017 and January 2018.

### PAED scale

The Pediatric Anesthesia Emergence Delirium (PAED) scale is a reliable tool to measure ED and involves five items: eye contact, purposeful actions, awareness of surroundings, restlessness, and inconsolability (Additional file 1). These five items are scored from 0 to 4. Patients with a total PAED score of ≥ 10 are defined as having an ED event [8].

### Translation and cross-cultural adaptation

Translation and cross-cultural adaptation processes were carried out in six stages, as recommended by the literature. In *Stage 1* (translation), the PAED scale was first translated into Spanish by three independent native Spanish anaesthesiologists who were fluent in English. In *Stage 2* (synthesis), the three translated versions were combined into one version; any disagreements were resolved by consensus under the supervision of three methodologists. In *Stage 3* (back-translation into English), three native English speakers who were fluent in Spanish and blinded to the purpose of the study independently performed back-translation. *Stage 4* (expert committee review) involved a consensus meeting to resolve any remaining problems, ambiguities, and discrepancies and to establish a prefinal Spanish version of the scale (all authors). In *Stage 5* (Pretesting), 54 paediatric anaesthesiologists and 47 anaesthesia recovery nurses in 22 health institutions in Chile were interviewed; they were asked about the importance and acceptability of the scale in clinical settings and to evaluate the scale’s wording accuracy and ease of understanding. In *Stage 6* (Integration), the answers of the pretesting stage were summarized, evaluated by all the authors, and integrated into a final version of the scale. Minor discrepancies were addressed based on consensus [12–14].

### Reliability of the Spanish version of the PAED scale

The reliability of the Spanish version of the PAED scale was evaluated in paediatric patients in the postanaesthetic period after major outpatient surgery. The scale was applied by previously trained observers (anaesthesiologists and/or PACU nurses), with each observer using a couple of measures at 0 min (the moment of recovery of consciousness) and 10 min after the recovery of consciousness in the postanaesthesia care unit (PACU). The scale was applied by two or three observers at the same time. The anaesthesiologist and/or nurse were blinded to each other’s score. Data were recorded in real time in a web-based ad hoc database (DPAP app) with synchronized clocks and were then encrypted once registered. We based all PACU management on the clinical conditions of the children and not on their PAED scores.

### Observer training

Both anaesthesiologists and nurses received one month of training. The training consisted of education sessions about ongoing research, paediatric anaesthesia emergence delirium, the use of the instrument (translated PAED scale), and the use of the electronic application (DPAP app). All observers independently assessed at least 50 patients who were not included in the study. In addition, nurses had to have at least three years of experience in postanaesthetic care, and anaesthesiologists had to have at least 3 years of experience.

### Study population

This study was conducted in compliance with the principles of the Declaration of Helsinki. The protocol of this study was reviewed and approved by the Institutional Review Board (Res Ext N0. 27,685/2016) and the Ethics Committee of the Universidad de La Frontera (File No. 018/2017). Written informed consent was obtained from parents/guardians by a research assistant. The inclusion criteria were as follows: children with an ASA physical status class of I, II or III, children who were cognitively intact, and children aged from 2–16 years who were undergoing elective outpatient surgery and general anaesthesia over a period of 7 months. The exclusion criteria were as follows: children with psychological, psychiatric or emotional disorders, children with developmental delays, children with neurological injuries, and children who needed sedative medication before induction.

### Anaesthesia

Children underwent general anaesthesia with total intravenous anaesthesia (TIVA) or sevoflurane anaesthesia (SEVO), as per the staff anaesthesiologist. Patients fasted for at least 6 h before the surgery but were allowed to drink clear fluids until 3 h before the surgery. No standardization was made for the methods of induction and maintenance of anaesthesia. A parent was present in the operating room (OR) for each induction if desired.

### TIVA induction and maintenance

After the placement of an IV cannula, anaesthesia was induced with a bolus of lidocaine (0.5–1 mg/kg) followed by target controlled infusion (TCI) anaesthesia using the Paedfusor model for propofol [15]. The desired effect site concentration was programmed by the staff anaesthesiologist. Remifentanil was maintained using an infusion of a fixed concentration solution of 20 μg/ml and was titrated as per the staff anaesthesiologist (0.2–0.5 μg/kg/min). For children in whom an IV cannula could not be placed, the use of inhalation induction with sevoflurane was not a contraindication to TIVA use. Conversion to TIVA after inhalation induction includes a slower loading dose administration or starting TCI at a lower target and slowly increasing it.

### SEVO induction and maintenance

Anaesthesia was induced with a mixture of 50% O2 in air by mask for 30 s followed by incremental increases in inspired sevoflurane (1–7%) until unconsciousness was achieved. IV access was obtained, and a bolus of lidocaine (0.5–1 mg/kg) and fentanyl (2 μg/kg) was administered. Anaesthesia was maintained by the titration of sevoflurane with 50% O2 in air as per the staff anaesthesiologist.

### Intraoperative anaesthetic management

Following the induction of anaesthesia, all subjects received a standard intraoperative IV analgesic medication of acetaminophen 15 mg/kg and ketoprofen 2 mg/kg and antiemetic prophylaxis of dexamethasone 0.15 mg/kg. In the SEVO group, supplementary doses of fentanyl (2 μg/kg) were administered after the induction dose until the end of surgery at the discretion of the attending anaesthesiologist.

### Statistical analysis

Demographic data are expressed as the mean ± standard deviation or percentages. Interobserver reliability was assessed using the Kappa [16] statistic. Internal consistency was estimated using Cronbach’s alpha test. A Cronbach’s alpha value ranging from 0.70 to 0.95 was considered adequate. The Bland‒Altman method was applied to calculate the difference and mean of measures. Data were analysed using Stata 14.0 software (version 14.0, Stata Corporation, College Station, TX). *P* < 0.05 was considered statistically significant. The sample size was estimated using the method of Tinsley [17], with the use of 5–10 subjects per item up to a total of approximately 300 patients.

## Results

### Scale

**Fig. 1:**
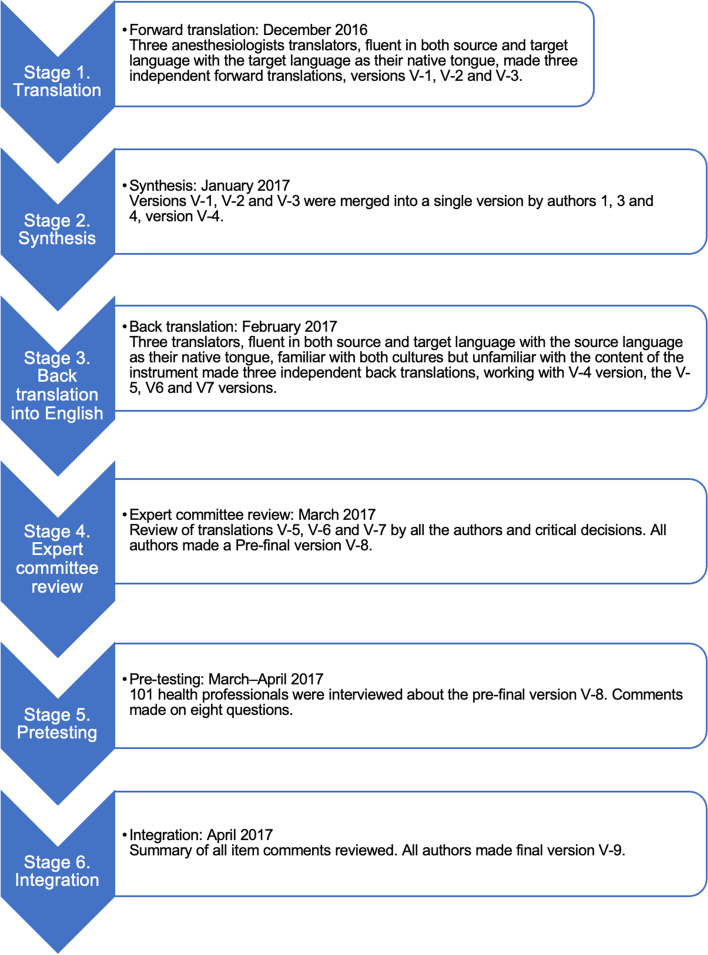
Translation and cross-cultural adaptation of the instrument

The translation and adaptation process are described in Fig. 1. The translated PAED scale can be seen in Additional file 1.

### Patients

Three hundred fifty-three consecutive children were enrolled in the study. Five children who met the inclusion criteria did not want to participate in the study. The demographic characteristics are summarized in Table 1. The PAED score ranged from zero to 19 (median 2, [IQR 0–5]).

**Table 1:** Demographic and perioperative characteristics presented as the mean ± standard deviation or counts (percentages)

Characteristic	Total population (*n* = 353)	Emergence Delirium (*n* = 32)	No Emergence Delirium (*n* = 321)
Age, years [min—max]	7.4 ± 3.22 [1.7–15]	5.9 ± 3.22 [2.2–9.5]	7.5 ± 3.22 [1.7–15]
Sex, female	115 (32)	12 (38)	103 (32)
Weight, kg	30.7 ± 14.23	24.1 ± 14.27	31.3 ± 14.23
ASA Physical Status			
I	219 (62)	22 (69)	197 (61)
II	131 (37)	10 (31)	121 (38)
III	3 (1)	0 (0)	3 (1)
Surgery			
Paediatric general surgery	159 (45)	8 (25)	151 (46)
Otorhinolaryngological surgery	116 (33)	22 (69)	94 (29)
Orthopaedic surgery	38 (11)	1 (3)	37 (12)
Ophthalmological surgery	37 (10)	0 (0)	37 (12)
Other	3 (1)	1 (3)	2 (1)
Anaesthesia			
TIVA	292 (83)	27 (84)	265 (83)
SEVO	61 (17)	5 (16)	56 (17)
Anaesthetic time, min	53.4 ± 24.26	49.7 ± 24.26	53.8 ± 24.04
Surgical time, min	45.1 ± 23.68	41.3 ± 23.68	45.5 ± 23.46
Awakening time, min	24.3 ± 13.36	18 ± 13.36	24.9 ± 13.35

### Reliability

Four anaesthesiologists and four anaesthesia recovery nurses administered the translated PAED scale (Additional file 1). The PAED score was compared 1753 times. Identical scores for ED were given in 67.9% of cases. Testing for interobserver reliability (Kappa coefficient) by observer is summarized in Table 2.

**Table 2:** Testing for interobserver reliability by Kappa coefficient

Pair of observers (numbers of comparisons)	Interobserver reliability	Agreement interpretation
	Precision	
	Kappa [95% CI]	
All (1753)	97.3%	Substantial
	**0.69** [0.60–0.77]	
Anaesthesiologist/Anaesthesiologist (142)	97.9%	Substantial
	**0.66** [0.30–1.00]	
Anaesthesia recovery nurse/Anaesthesia recovery nurse (642)	97.8%	Almost perfect
	**0.79** [0.68–0,90]	
Anaesthesiologist / Anaesthesia recovery nurse (969)	96.9%	Substantial
	**0.59** [0.46–0.72]	

**Fig. 2:**
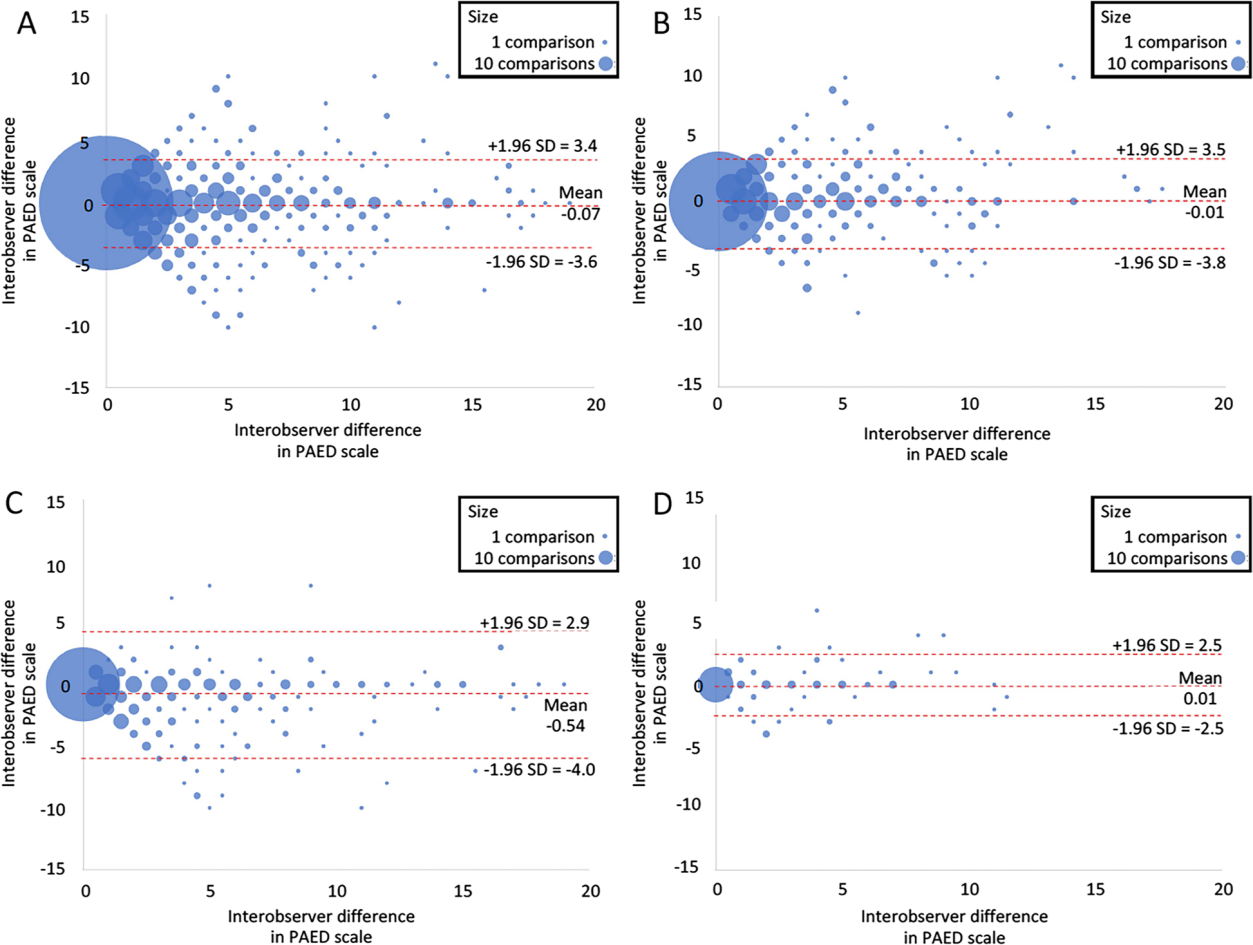
Bland‒Altman plot showing the interobserver agreement for the DPAP scale. Bland‒Altman plots of the mean DPAP score against the difference between the observers. The dotted red lines represent the mean ± 1,96 standard deviations (SDs) of the differences. Each bubble represents the count of the comparisons. The largest bubble in (**A**) represents 915 comparisons matching a PAED score of zero (0). **A** All comparisons. **B** Comparisons between anaesthesiologists and nurses. **C** Comparisons among nurses. **D** Comparisons among anaesthesiologists

In Bland‒Altman analysis, 95.7% of comparisons were included between ± 1,96 SD (Fig. 2). Overall, the Cronbach’s alpha value was 0.91, indicating strong internal consistency.

### ED incidence

**Fig. 3:**
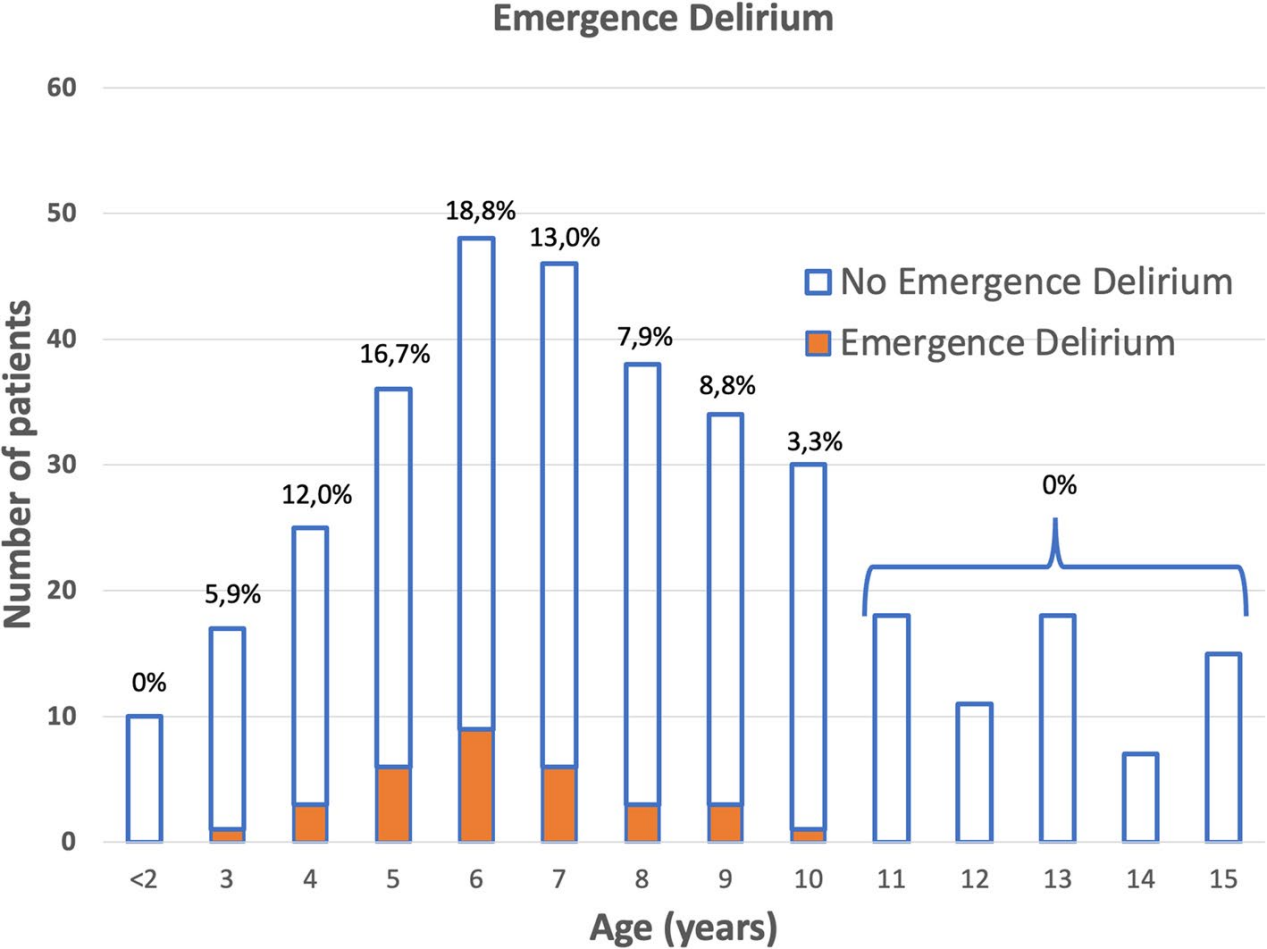
Incidence of emergence delirium by age

The global incidence of ED was 9.1%. The incidence was higher in the younger age groups, with no ED in children under 2 years of age or in those over 10 years of age (Fig. 3).

## Discussion

To the best of our knowledge, this is the first translation and cultural adaptation study of the PAED scale into Spanish. Our study determined the reliability of the Spanish translation of the PAED scale in Chilean children. We found a good level of agreement among anaesthesiologists and/or nurses (Table 2), with a Kappa value considered to be at least substantial [16]. Assessing this scale among anaesthesiologists and nurses allows us to use it interchangeably among these professionals. Instruments require adaptation for use in a different country with both a different culture and a different language [13, 14]. Despite the relevant problem of ED, the PAED scale has only been validated and adapted into Danish [18].

The incidence of ED in paediatric patients ranges between 5 and 50% [2–4, 18]; we found an incidence of 9.1%. Although ED has a low incidence, we should emphasize that our study population was a selected population. It is possible that when applying the scale for patients who are subjected to longer-term surgeries or emergency surgeries, this incidence will vary and increase. ED was detected in preschool and child patients, with a higher incidence among children between four and seven years of age. This is consistent with what has been published in the literature with respect to the age distribution of ED [19–21].

To carry out the quantitative stage and decrease the probability of bias, the decision was made to create a web-based electronic application that would be available for electronic devices (mobile phones, tablets, desktop computers); therefore, each observer could access and record the measurement taken for a patient in real time while being blinded to the rest of the patient’s clinical conditions and to the measurements that another observer was making using the same application at the same time. The electronic application only allowed access to the patient's identification and the respective scale. It also did not provide the final score of the scale. All the above was considered to make the measurement have as little bias as possible.

Test–retest reliability was not assessed. Due to the nature of the clinical conditions evaluated, the patients’ conditions were highly variable in a minimum unit of time, which made it a difficult analysis. We could have used video clips of children to test the test–retest reliability [22], but we did not realize this until the study was very advanced.

Regardless of the standardized training provided for the observers, the different comparison groups did not perform the same. It is difficult to determine the cause of why there were differences in agreements between groups. The best performance was obtained by nurses, possibly because they are used to working as a team, and they do not face patients with ED alone, so their criteria are very similar. Anaesthesiologists, on the other hand, are more solitary professionals, each with their own interpretation of clinical scenarios. Another element that could influence the results is the fact that while the number of nurses and anaesthesiologists who participated was equitable (four anaesthesiologists and four nurses), the number of measurements made by each group was not the same, and therefore the vast majority of comparisons were based on measurements made by nurses, as shown in Table 2, which shows that 90% of the measurements involved a nurse and 63% of the measurements involved anaesthetists in the total comparisons. Despite the above, the agreement between the different professionals was very good at the time of applying the scale.

The PAED scale is the only validated scale that exists to assess postanaesthetic delirium in paediatric patients undergoing general anaesthesia [23]. Since its creation, it has been used in numerous publications that have endeavoured to address this complex issue in children and is currently considered the standard for diagnosing ED [24]. This observational study demonstrated that the translated and cross-culturally adapted Spanish version of the PAED scale (Chile) is a reliable instrument to assess ED in the postanaesthetic period in Chilean children. This tool provides clinicians with an objective method of ED assessment, which may offer an opportunity to detect and treat ED in children undergoing general anaesthesia in our setting. This culturally adapted version Spanish version of the PAED scale was translated for use in the Chilean population; nevertheless, it can be used as a basis for future adaptations in other Spanish-speaking countries.

## Conclusions

The use of validated scales allows the generation of standardized measurements that can be compared among different settings. However, each setting contains unique characteristics that must be considered when choosing the different measurement scales. The translated and culturally adapted Spanish version of the PAED scale has been shown to perform well both in the measurements made by anaesthesiologists and nurses. The PAED scale has not been previously translated or adapted to the Spanish language, so this tool can be the basis for the generation of new knowledge related to the mechanisms, biology and treatment of postoperative delirium in paediatric patients in Spanish-speaking countries.

## Supplementary Information

**Additional file 1.** 

**Additional file 2.** 

## Data Availability

The datasets generated and analysed during the current study are available in the DPAP_Data.xlsx file as a supplementary file.
