# Supplementary material for: Spanish version of the Pediatric Anesthesia Emergence Delirium scale: translation and cross-cultural adaptation
Source: BMC Anesthesiol. 2022 Nov 14;22:349. doi: 10.1186/s12871-022-01893-1 (PMC9661762; doi:10.1186/s12871-022-01893-1)
Supplement: Supplementary file 1 — Additional file 1. [file 12871_2022_1893_MOESM1_ESM.docx]

Additional file 1

DPAP scale

| **Point** | **Description** | **Not at All** | **Just a Little** | **Quite a bit** | **Very Much** | **Extremely** |
| --- | --- | --- | --- | --- | --- | --- |
| 1 | The child makes eye contact with the caregiver | 4 | 3 | 2 | 1 | 0 |
| 2 | The child’s actions are purposeful | 4 | 3 | 2 | 1 | 0 |
| 3 | The child is aware of his / her surroundings | 4 | 3 | 2 | 1 | 0 |
| 4 | The child is restless | 0 | 1 | 2 | 3 | 4 |
| 5 | The child is inconsolable | 0 | 1 | 2 | 3 | 4 |

DPAP translated and adapted to Spanish scale

| **Descripción** | **No en lo absoluto** | **Sólo un poco** | **Bastante** | **Mucho** | **Extremadamente** |
| --- | --- | --- | --- | --- | --- |
| El niño(a) hace contacto visual con el cuidador | 4 | 3 | 2 | 1 | 0 |
| Las acciones del niño(a) tienen un propósito. | 4 | 3 | 2 | 1 | 0 |
| El niño(a) está consciente de su entorno. | 4 | 3 | 2 | 1 | 0 |
| El niño(a) está inquieto(a). | 0 | 1 | 2 | 3 | 4 |
| El niño(a) está inconsolable. | 0 | 1 | 2 | 3 | 4 |
